# A comparison of curated gene sets versus transcriptomics-derived gene signatures for detecting pathway activation in immune cells

**DOI:** 10.1186/s12859-020-3366-4

**Published:** 2020-01-28

**Authors:** Bin Liu, Patrick Lindner, Adan Chari Jirmo, Ulrich Maus, Thomas Illig, David S. DeLuca

**Affiliations:** 1 Hannover Medical School, Biomedical Research in Endstage and Obstructive Lung Disease Hannover (BREATH), German Center for Lung Research, Carl-Neuberg-Straße, Hannover, 30625 Germany; 2 Institute of Technical Chemistry, Leibniz University of Hannover, Callinstraße 5, Hannover, 30167 Germany; 3 Hannover Unified Biobank, Hannover Medical School, Feodor-Lynen-Straße, Hannover, 30625 Germany; 4 Division of Experimental Pneumology, Hannover Medical School, Feodor-Lynen-Straße 21, Hannover, 30625 Germany; 5 Department of Pediatric Pneumology,Allergology and Neonatology, Hannover Medical School, Carl-Neuberg-Straße 1, Hannover, 30625 Germany

**Keywords:** Transcriptome, Gene signature, Gene set

## Abstract

**Background:**

Despite the significant contribution of transcriptomics to the fields of biological and biomedical research, interpreting long lists of significantly differentially expressed genes remains a challenging step in the analysis process. Gene set enrichment analysis is a standard approach for summarizing differentially expressed genes into pathways or other gene groupings. Here, we explore an alternative approach to utilizing gene sets from curated databases. We examine the method of deriving custom gene sets which may be relevant to a given experiment using reference data sets from previous transcriptomics studies. We call these data-derived gene sets, “gene signatures” for the biological process tested in the previous study. We focus on the feasibility of this approach in analyzing immune-related processes, which are complicated in their nature but play an important role in the medical research.

**Results:**

We evaluate several statistical approaches to detecting the activity of a gene signature in a target data set. We compare the performance of the data-derived gene signature approach with comparable GO term gene sets across all of the statistical tests. A total of 61 differential expression comparisons generated from 26 transcriptome experiments were included in the analysis. These experiments covered eight immunological processes in eight types of leukocytes. The data-derived signatures were used to detect the presence of immunological processes in the test data with modest accuracy (AUC = 0.67). The performance for GO and literature based gene sets was worse (AUC = 0.59). Both approaches were plagued by poor specificity.

**Conclusions:**

When investigators seek to test specific hypotheses, the data-derived signature approach can perform as well, if not better than standard gene-set based approaches for immunological signatures. Furthermore, the data-derived signatures can be generated in the cases that well-defined gene sets are lacking from pathway databases and also offer the opportunity for defining signatures in a cell-type specific manner. However, neither the data-derived signatures nor standard gene-sets can be demonstrated to reliably provide negative predictions for negative cases. We conclude that the data-derived signature approach is a useful and sometimes necessary tool, but analysts should be weary of false positives.

## Background

With the advent of high-throughput sequencing technology, transcriptome data sets are being generated on a massive scale. Differential expression analyses produce long lists of genes, requiring summarization approaches to allow for the biological interpretation of the results. Gene Set Enrichment Analysis (GSEA) [1], Over-Representation Analysis (ORA) and Gene Set Analysis (GSA, also referred to as Pathway Analysis) have been developed to this end. These methods rely on catalogues of gene sets which are associated with various biological processes, relying either on literature or high throughput experimentation. By applying statistics such as the Mann-Whitney-Wilcoxon Test, Fisher’s Exact Test [2] or the Kolmogorov-Smirnov statistic [1], these methods enable one to interpret the relevance of a biological process in a given experiment. The curated gene sets are archived in a range of biological pathway databases including the Gene Ontology (GO) [3], the KEGG (Kyoto Encyclopedia of Genes and Genomes) Pathway database [4] and The Reactome Knowledgebase [5].

Although GSEA, ORA and GSA have been widely adopted to interpret the results of transcriptomics studies, their value is limited by the number of curated gene sets available to the researchers. In many cases, researchers may fail to find curated gene sets from such databases best describing the complex immunological process they are most interested in. In other cases, curated gene sets may reflect cell-type specificity, and could involve multiple cell types. This creates a limitation in applicability to expression based studies in which a single cell type is profiled.

Meanwhile, the construction of repositories for archiving transcriptomic data sets enables one to access high-quality data generated by previous studies. Gene Expression Omnibus (GEO) from the National Center for Biotechnology Information (NCBI) [6] and ArrayExpress from European Molecular Biology Laboratory (EMBL) [7, 8] are two prominent examples among many. Given this wealth of resources, we examine a data-derived signatures approach. We evaluated the simple methodology of testing whether a given biological process is activated in a target data set by deriving a relevant gene signature from a previous transcriptomics experiment and testing the presence of that signature in the target data set. Deriving gene sets directly from previous transcriptomics experiments has several precedents, for example portions of MSigDB [9, 10]. Here, we focus our analysis in the context of immune cells. We evaluate several statistical approaches to detecting the activity of a gene signature in a target data set, some of which are previously used in gene set enrichment analysis such as Wilcoxon Test, and Fisher Exact Test, as well as a novel expression concordance score test. We compare the performance of the data-derived gene signature approach with comparable GO term gene sets across all of the statistical tests.

## Methods

All the tests in this section were processed using R (Version 3.2.3) on platform x86_64-pc-linux-gnu (64-bit) [11] embedded in RStudio (Version 1.1.463) [12].

### Data source

We downloaded 25 immune-related Series (GSE) from GEO repository in the Series Matrix File form, 22 of which are describing significantly activated biological processes and will be used for generation of the signatures or true positive targets for validation. The remaining three were used as negative targets for assessing specificity. T Nikolic et al. supplied the analyzed data of their publication [13], which systematically profiled the differentially expressed genes (DEGs) between tolerogenic dendritic cells (tol-DCs) and non-modulated mature inflammatory dendritic cells (mDCs). A brief description of each data set is given in Table 1.

**Table 1:** Transcriptomics data sources

ID	Experiment design	Experimental Organism	Contributor
tolerogenic_DC	Tolerogenic DC	Homo Sapiens	T Nikolic et al. [13]
GSE17721	Tolerogenic DC	Mus Musculus	I Amit et al. [14]
GSE18921	Tolerogenic DC	Homo Sapiens	H Torres-Aguilar et al. [15]
GSE5099	Monocytes differentiation	Homo Sapiens	FO Martinez et al. [16]
GSE8286	Monocytes differentiation	Homo Sapiens	H Liu et al. [17]
GSE111475	B cell activation	Homo Sapiens	K Miyawaki et al.
GSE116999	B cell activation	Homo Sapiens	DT Avery et al. [18]
GSE51587	B cell activation	Homo Sapiens	LJ Berglund et al. [19]
GSE54017	B cell activation	Homo Sapiens	A Shimabukuro-Vornhagen et al. [20]
GSE29797	T cell activation	Mus Musculus	Yang K et al. [21]
GSE112899	T cell activation	Homo Sapiens	Sousa IG et al. [22]
GSE60235	T cell activation	Homo Sapiens	Ye CJ et al. [23]
GSE73213	T cell activation	Homo Sapiens	LaMere SA et al. [24, 25]
GSE111789	Eosinophils cytokine response	Homo Sapiens	Khoury P et al. & Gadkari M et al. [26, 27]
GSE112010	Eosinophils cytokine response	Mus Musculus	Fairfax KA et al. [28]
GSE128027	Eosinophils cytokine response	Homo Sapiens	Nelson RK et al. [29]
GSE104152	Naive to Th17 differentiation	Mus Musculus	Mohammad I et al. [30]
GSE113889	Naive to Th17 differentiation	Homo Sapiens	Tangye S et al. (Accession: GSE113889)
GSE118974	Naive to Th17 differentiation	Homo Sapiens	Tripathi SK et al. [31]
GSE140443	Naive to Th17 differentiation	Mus Musculus	Gehrmann U et al. (Accession: GSE140443)
GSE110446	NK IL12	Homo Sapiens	Costanzo MC et al. [32]
GSE24791	NK IL12	Homo Sapiens	Campbell AR et al. [33]
GSE63038	NK IL12	Homo Sapiens	de Carvalho EG et al. (Accession: GSE63038)
GSE87290	PBMC LPS	Homo Sapiens	Lin J et al. [34]
GSE22248	PBMC LPS	Homo Sapiens	Pena OM et al. [35]
GSE9916	PBMC LPS	Homo Sapiens	Wong HR et al. [36]
GSE101710	Negative data	Homo Sapiens	Zapata HJ et al. [37]
GSE110223	Negative data	Homo Sapiens	Vlachavas EI et al. [38]
GSE21045	Negative data	Homo Sapiens	Landolin JM et al. [39]

### Signature generation

We consider a signature to be a collection of genes whose expression changes in association with a specific cellular process. The signatures to be detected were generated by the following two strategies:

**1) Generation of data-derived signature and target data sets:** We downloaded 28 immune-related Series (GSE) from the GEO repository in the Series Matrix File form, 25 of which describe activated biological processes. T Nikolic et al. supplied the analyzed data of their publication [13], which systematically profiled the differentially expressed genes (DEGs) between tolerogenic dendritic cells (tol-DCs) and non-modulated mature inflammatory dendritic cells (mDCs).

To access specificity, negative target data sets are required. The main approach we take is to simply consider for a given immunological process, the target data sets of the remaining immunological processes in our study to be negative cases. We also provide an alternative approach to defining negative cases in which the control samples from Series GSE101710, GSE110223, and GSE21045 were randomly selected and equally distributed into two different groups, representing pseudo-phenotypes for differential expression.

Both the data-derived signatures and target datasets are based on differential expression (DE) analysis. The Differential expression of microarray data were analyzed in R using limma (Version 3.26.9) [40]. The RNA-seq data of GSE112899, GSE73213, GSE111789 and GSE128027 were analyzed in R using DESeq2 package (Version 1.24.0) [41]. *P*-values of the DE (differential expression) analysis were corrected for multiple tests using the q-value method [42].

For each immunological process in our study, we obtained multiple GEO series, one of which we selected for signature generation and the remaining experiments represent the target data sets. We then iterated the target series, and selected each of them a signature set, such that each experiment was considered to be a target set and a signature set at one point. We also assessed the impact of selecting the “best” experiment as the signature data to determine whether being selective in choosing the signature set could be beneficial. Here the “highest quality” is defined by making a judgment based on the sample size, platform, and specifics about the experimental design.

**2) Selection of curated gene sets:** For each of the relevant cellular processes captured by the data-derived signature, we searched the literature and gene set databases (GO, KEGG) for appropriate gene sets. We included a literature-based tolerogenic DC (dendritic cell) signature as proposed by C Orabona et al. [43]. No comparable tolerogenic DC signatures were available in the KEGG or GO databases. We also merged the annotation list of GO term Positive Regulation of Monocyte Differentiation (GO:0045657) and Negative Regulation of Monocyte Differentiation (GO:0045656) as the curated gene set for Monocyte differentiation and Positive Regulation of B Cell Activation (GO:0050871) and Negative Regulation of B Cell Activation (GO:0050869) as the curated gene set for B cell activation. GO:2000417 Negative Regulation of Eosinophil Migration and GO:2000418 Positive Regulation of Eosinophil Migration was merged for detection of the Eosinophil cytokine response. GO:0032824 and GO:0032825 (Negative/Positive Regulation of Natural Killer Cell Differentiation) together as a merged list for NK (natural killer) IL12 (interleukin) stimulation and GO:0034142 (Toll-Like Receptor 4 Signaling Pathway) for PBMC (peripheral blood mononuclear cell) to LPS (lipppolysaccride) response. The curated data set for the detection of Naive to Th17 differentiation derived from a merged list of GO:0050868 and GO:0050870 (Negative/Positive Regulation of T helper-17 cell differentiation) and T cell activation described by merging the GO annotation of GO:0050868 and GO:0050870 (Negative/Positive Regulation of T cell activation). The list of curated gene sets utilized as the signature in this approach is found in Table 2.

**Table 2:** Currated gene sets

Name	Description	Process
GO:0050868	Negative regulation of T cell activation	Tcell_activation
GO:0050870	Positive regulation of T cell activation	Tcell_activation
GO:2000320	Negative	Regulation of T helper-17 cell differentiation
GO:2000321	Positive	Regulation of T helper-17 cell differentiation
GO:0032824	Negative regulation of natural killer cell differentiation	NK_IL12
GO:0032825	Positive regulation of natural killer cell differentiation	NK_IL12
GO:2000417	Negative regulation of eosinophil migration	Eosinophils_cytokine_response
GO:2000418	Positive regulation of eosinophil migration	Eosinophils_cytokine_response
GO:0045657	Positive regulation of Monocyte Differentiation	Monocyte_Macrophage_differentiation
GO:0045656	Negative regulation of Monocyte Differentiation	Monocyte_Macrophage_differentiation
GO:0050871	Positive regulation of B cell activation	Bcell_activation
GO:0050869	Negative regulation of B cell activation	Bcell_activation
GO:0034142	Toll-like receptor 4 signaling pathway	PBMC_LPS
tolerogenic DC signature	Tolerogenic DC signature [43].	Tolerogenic_Dendritic_cells

### Signature detection

We would like to know whether a cellular process represented by a signature has been deferentially regulated in a target experiment. To detect the presence of the signatures in the target data sets, we applied the following alternative methods:

**1) The Mann-Whitney-Wilcoxon Enrichment Test:** We used Mann-Whitney-Wilcoxon Test in the same manner found in the PANTHER [2] webtool. Genes are ranked by their fold change in the target data set. The test is then performed between the ranks of the signature genes versus the ranks of the non-signature genes. *P*-values are provided by the wilcox.test() function from R stats package [44].

**2) Fisher’s Exact Overrepresentation Test:** The Fisher’s Exact Test is applied in a comparable fashion to tool such as PANTHER [2]. The test is based on a contingency table comparing the differential expression status (DE or not DE) vs the signature status (in signature or not in signature). A demonstration of the contingency table is available in Table 3 below. *P*-values were generated by the fisher.test() function from R stats [44].

**Table 3:** A comparison of the contingency table for Concordance Fisher’s Exact (above) and Fisher’s Exact Overrepresentation (below)

	Genes matched in regulation direction	Genes not matched in regulation direction	Total
In signature	*x*	*m*−*x*	*m*
Not in signature	*k*−*x*	*n*−(*k*−*x*)	*n*
Total	*k*	*m*+*n*−*k*	*m*+*n*
	Differentially expressed genes	Not differentially expressed genes	Total
In signature	*x*	*m*−*x*	*m*
Not in signature	*k*−*x*	*n*−(*k*−*x*)	*n*
Total	*k*	*m*+*n*−*k*	*m*+*n*

**3) Correlation Permutation:** This method is a quantitative permutation test based on Spearman’s rank correlation. This correlation is calculated for the signature genes between their ranks in the signature and their ranks in the target data set based on fold change. In performing permutations, we randomly selected a gene set with the same length as the signature the set of genes common to the two data sets. The Spearman correlation was calculated for each of 10,000 permutation, giving an empirical distribution from which p-value was derived. The sign of the correlation represented the direction of the signature in the target set.

**4) Concordance Permutation:** The signature concordance Test is a semi-quantitative permutation test based on the regulation directions. For the test, a concordance score is defined as:

1 $$ concordance\ score\ = \frac{N_{signature\ genes\ matching\ in\ regulation\ directions}}{N_{genes\ in\ the\ signature}}  $$

where *N* refers to the number of genes.

The concordance score of the signature was calculated using Equation 1. A total of 10,000 permutations were performed to generate an empirical null distribution. In each permutation, we randomly selected a gene set with the same length as the signature from all the genes which are in common between the signature-generating platform and the target data set platform and record the concordance score. *P*-values are derived from the test statistic and the empirical null distribution. The signature direction is determined by whether the concordance score of the signature is greater or less than the mean of permuted scores.

**5) Concordance Fisher’s Exact Test:** As in the previous method, this method is also based on the concordance of the fold change direction, however with a different strategy for deriving a p-value. Here, we derive a p-value using the Fisher Exact Test based on the contingency table in Table 3.

It should be noted that the first two tests (overrepresentation and enrichment) consider the gene signature to be an unordered set of genes, and the resulting test does not provide a direction as to the regulation of the biological process. This is equally applicable to gene signatures and currated gene sets. However, the remaining correlation and concordance tests also provide a direction, and we considered correct direction to be a requirement for true positive / true negative results when accessing the accuracy. Furthermore, these tests require a ranking of the genes in the gene signature, which are not available for currated gene sets, and therefore these tests were not applied to the curated gene sets.

*P*-values of all the five methods were calculated according to the aforementioned description. ROC (receiver operating characteristic) curves were generated by changing the confidence level threshold (the threshold for *p*-values) for the logistic classification of whether the signature is presented in each target set. The AUC (area under the curve) of each condition was calculated for the comparison.

## Results

**Fig. 1:**
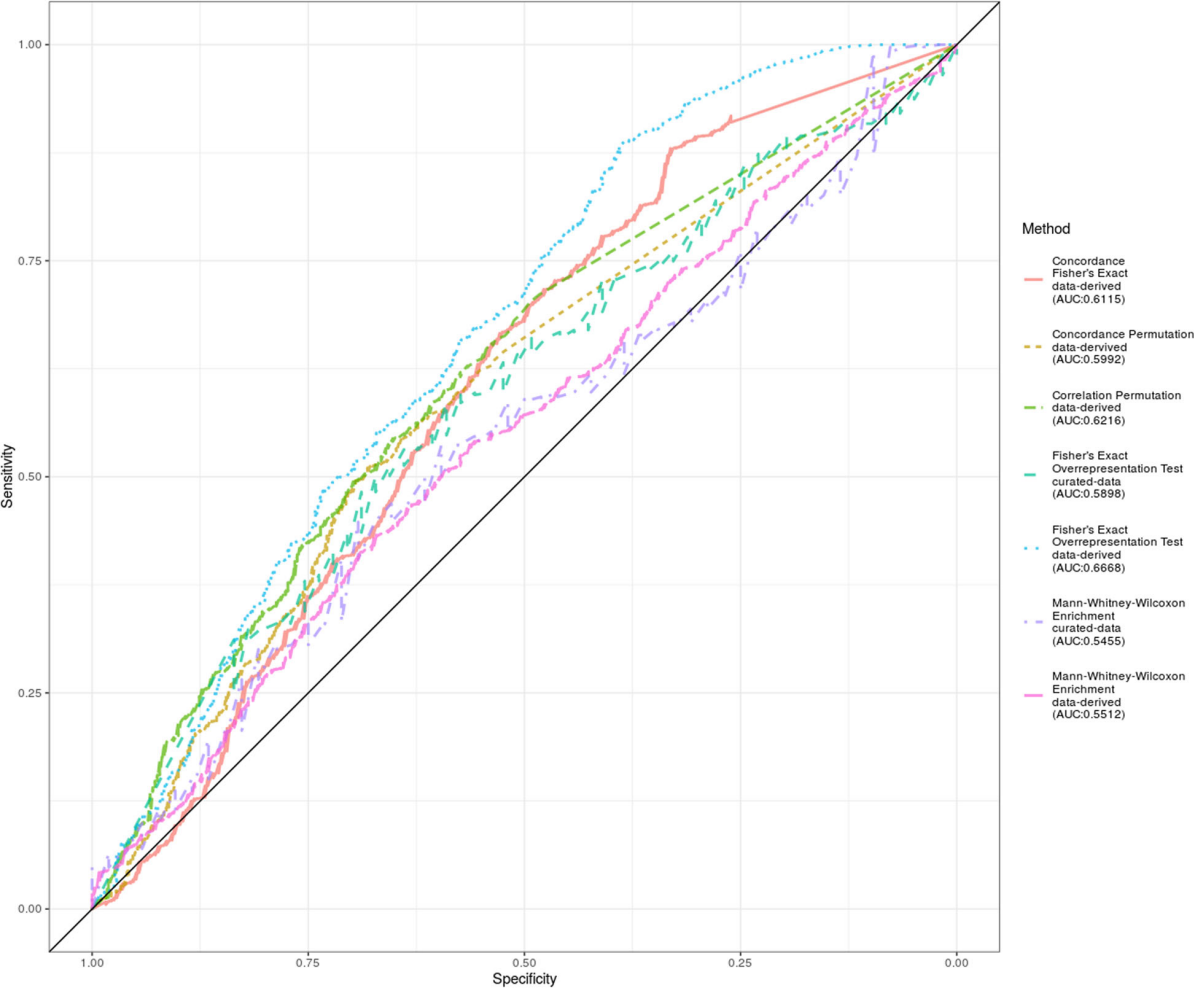
ROC curves for the application of the selected statistical methods on the detection of signature. The ROC curves described the performance of Fisher’s Concordance Test, Correlation Permutation Test, Concordance Permutation Test and Man-Whitney-Wilcoxon Test in the detection of both data-derived and curated-gene set signatures

We applied all the five methods on data-derived signatures to detect the presence of the signatures in the target data sets and to evaluate the performance of the methods on data-derived signatures. The ROC curves are summarized in Fig. 1, with AUC under each condition labelled separately. The full table of *p*-values for each target signature combination is provided in the supplement [Additional file 1].

The data derived signature tests performed more favorably than the curated gene set definitions, with the best performance being that of the Fisher Exact Overrepresentation Test for the data derived signature (AUC = 0.67). The Mann-Whitney-Wilcoxon Enrichment test performed poorly (AUC = 0.55) for both data-derived and curated gene sets.

While the ROC curve is useful for summarizing performance using a sliding threshold, given that these scores are based on *p*-values, it makes sense to example the specific ubiquitous threshold of 0.05. The sensitivity and specificity values for an alpha threshold of 0.05 are summarized in Table 4:

**Table 4:** The sensitivity and the specificity of the five methods at an alpha cutoff of 0.05

Signature Generation	Data-derived signature		Curated gene set	
Method	Sensitivity	Specificity	Sensitivity	Specificity
Mann-Whitney-Wilcoxon Enrichment	0.7655	0.3098	0.25	0.7292
Fisher’s Exact Concordance	0.7977	0.3720	–	–
Fisher’s Exact Overrepresentation	0.8981	0.1014	0.5246	0.6229
Correlation Permutation	0.7278	0.4517	–	–
Concordance Permutation	0.7424	0.3995	–	–

### Robustness analysis

**Fig. 2:**
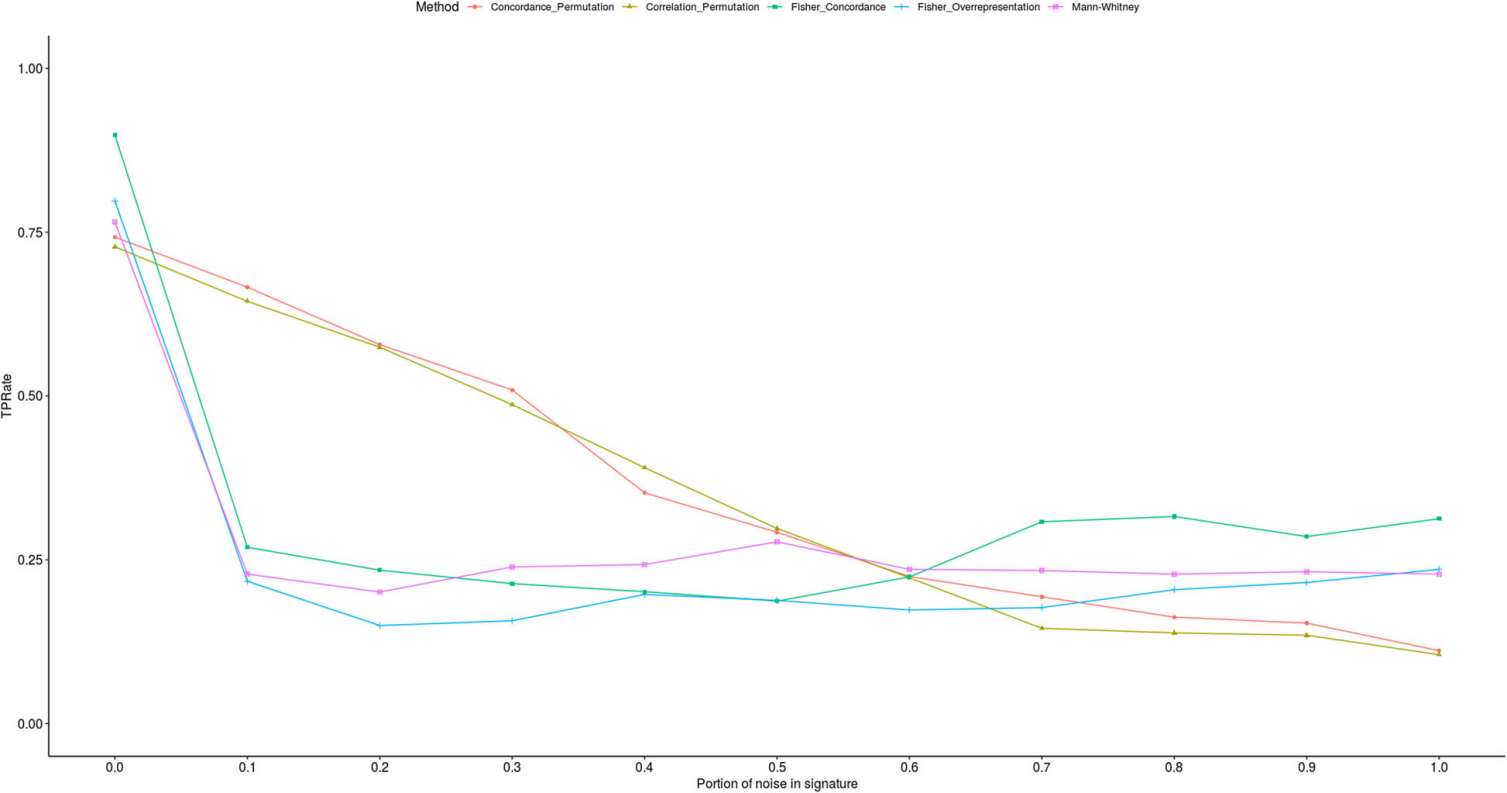
True Positive Rate as a function of noise added to the signatures. Noise was added to the signature by swapping the proportion of genes indicated on the x axis with the least significant genes in the signature’s differential expression experiment

We assessed the behavior of these statistics in the presence of increasing noise in the signatures. This was done by replacing a batch of the most significant with the least significant genes, and iteratively increasing the batch size. The effect of noise on the True Positive Rate is showing in Fig. 2. The full table of results is provided in the supplement [Additional file 2].

The robustness analysis shows that the analytical statistics do show a sharp decline in True Positive Rate with the introduction of only 10 percent noise. In contrast, the permutation-based methods show a more gradual effect with increasing noise.

**Fig. 3:**
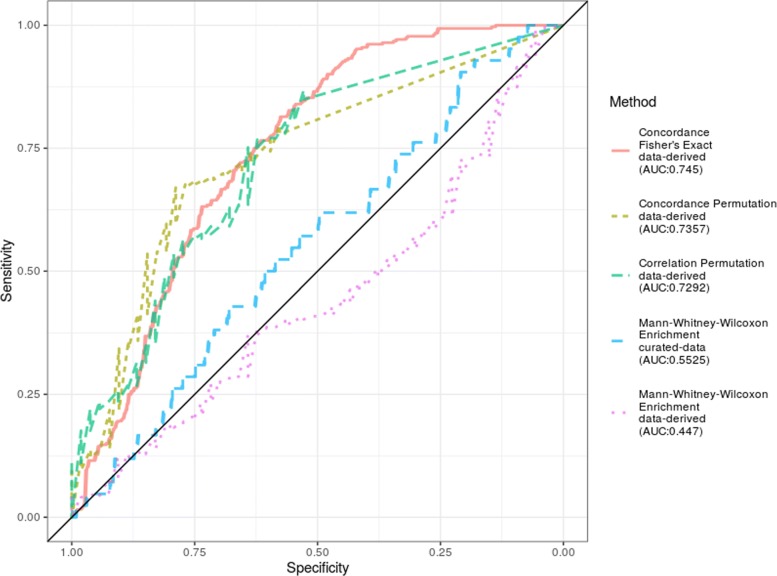
ROC curves when selecting high quality experiments to define signatures Here, not every experiment was evaluated as a signature data set. Instead, for every immune process only one experiment was chosen to generate the signature based on quality considerations such as number of samples, experimental design, and platform.

Among the various experiments in our study, we can expect various levels of “noise” also in the sense that the quality of the experiments could be quite heterogeneous. We have accessed the effect of being selective in choosing our signature-generating data sets and calculated the performance when using only the highest quality experiments for generating the signatures (based on sample size, experimental design, platform). The result is an increase in AUC (Fig. 3). This selectivity boosts the AUCs above 0.72 for several statistics, indicating the value of basing signatures on high quality experiments.

### Specificity analysis

**Fig. 4:**
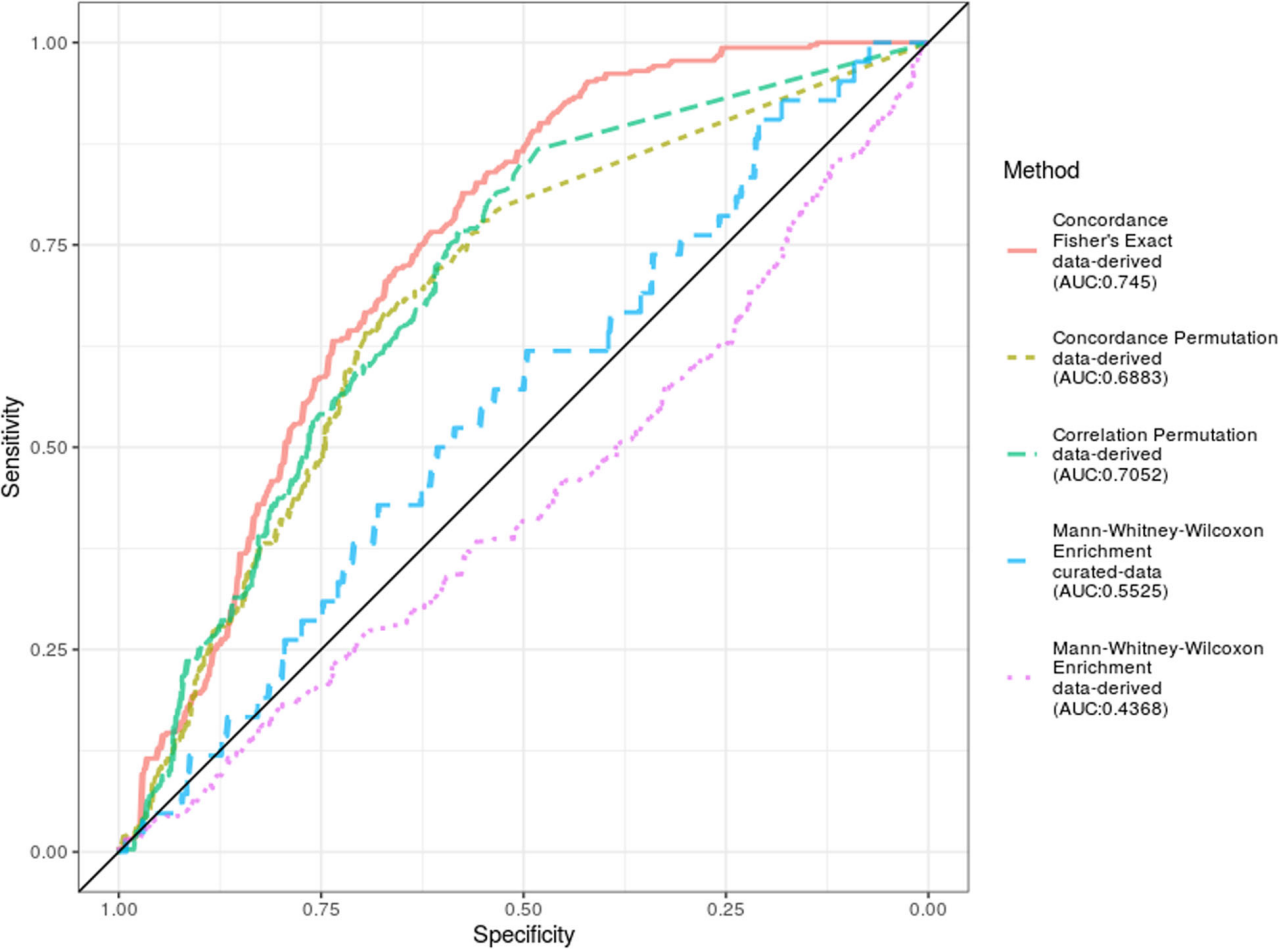
ROC curves when pseudo-phenotypes are used as negative cases In this alternative definition of negative cases, control samples were randomly assigned to each of two categories and a differential expression analysis was performed, thus generating a negative target dataset

Faced with the need to define a set of target data sets as negative cases, we have opted to consider for a given signature of an immunological process the negative cases to be the target data sets for the other immunological processes which we have collected. However, motivated by the fact that there will be overlapping genes involved in the immunological processes we include here (e.g. genes that are upregulated both during B cell and T cell stimulation), we also provide an alternative definition of negative cases. We have taken control samples from three additional studies and generated a series of random combinations of samples into pseudo-phenotypes for differential expression analysis. Using this definition of negative samples, we observe the ROC curves in Fig. 4. This type of assessment does lead to higher AUC scores due to the fact that the top DE genes in the pseudo phenotpyes are completely independent of the processes at play in the signature experiments.

To examine the sources of non-specific signature genes, we identified genes that deferentially expressed across most of the signatures. We selected genes whose median absolute fold change was greater than 1 across all the signatures. These genes are provided in supplement [Additional file 3]. Performing a GO enrichment analysis with PANTHER, we see that these genes are common to a wide range of immunological processes [Additional file 3].

## Discussion

We have evaluated methods for detecting the activation or deactivation of immunological processes within target differential expression experiments. We compared a strategy based on gene signatures derived from previous transcriptome experiments with the approach more commonly taken using curated gene sets. The results show that the use of such “custom” gene signatures is a valid approach, despite the fact that they are derived from single experiments, whereas curated gene sets can be based on many sources of information. Overall both methods tend to produce high rates of false positives.

This observation of an abundance of false positives is consistent with previous studies. Tarca et al have taken a similar approach to evaluating their gene set enrichment tool along with other published methods by defining a test data set that consisted of previous DE experiments [45]. They generated signatures for various cancer types and tested them in matching and mismatching cancer target sets. The false positive results were so prevalent that they took the strategy of using the rank of the correct target set in the list of all tested sets to define their performance metric. In other words, the best method was the one that had the fewest false positives of higher rank than the correct target.

Although we evaluate the performance of these methods using AUC, it is important to note that in practice a p-value cutoff of 0.05 is typically used when deciding whether a gene set is differentially expressed. At this cutoff the data-derived signature method exhibits high sensitivity and low specificity. In practice, this method would benefit from a more stringent p-value threshold.

When comparing the various statistical tests among themselves, the Mann-Whitney-Wilcoxon notably under-performs. For the data-derived signatures, at the p-value threshold of 0.05, the performance is characterized by poor specificity for data-derived signatures and poor sensitivity for curated gene sets. Given the large size of the gene signatures, the test is perhaps very sensitive to even small amounts of bias, resulting in many false positive calls. For the smaller curated gene sets, the method seems to be somewhat more appropriate.

When applying the data-derived gene signatures, we tested two groups of statistics: ones which utilize the direction of the fold change in the signature data set, and those which use the signatures simply as a set without regard to the direction of change in expression. These results are somewhat ambiguous as to whether there is an advantage to using this additional information, given that one of the two techniques which do not use it performed quite well – namely the Fisher Exact Overrepresentation.

In principle, the a compendium of data derived gene signatures could be generated exhaustively for all of GEO. The MSigDB has taken steps in this direction with for example the C7 collection of immunological signatures. These collections do contain genes and their change in direction, however information is missing concerning how many genes are in common between the signature-generating platform and the target data set platform - a discrepancy which will only increase over time with the introduction of new technologies. An additional issue is that these signatures are limited to 200 genes per direction, which is smaller than our data-derived signatures and in our hands shortening the signatures to this length decreases accuracy. There is also a cost trade off between running an “everything against everything” analysis, as is the case in C7 versus careful manual differential expression analysis by an expert, involving quality control steps and consideration of covariates, batch effect, etc. The former does allow for hypothesis free analysis, however at the expense of many inapplicable tests that reduce power when it comes to multiple test correction.

## Conclusion

In conclusion, the data-derived gene signature approach is a valid and useful tool for inferring the presence and absence of immunological processes in transcriptome datasets. The approach makes valuable use of previously published experiments, and can be carefully tailored to ensure that the most relevant comparisons are made when using a hypothesis driven technique. The accuracy is reasonable when compared to the gene set based approach, but both approaches are prone to false positives. The weakness in widely used gene set based approaches is overlooked, perhaps due to the difficulty in producing ground truth information, but this is an issue that must be addressed to improve the interpretation of transcriptome experiments.

## Supplementary information

**Additional file 1** All Evaluation Results Description: Contains the results for all combinations of signatures, target data sets, and alternative statistical methods. Each sheet represents a statistical method. Each row contains a signature, a target and a series of values that are specific to each of the different approaches.

**Additional file 2** Robustness Analysis Description: Results of the robustness analysis when adding noise to the signature. “Portion” refers to what percentage of genes were swapped and the remaining columns are the metrics and outcomes for the various statistical approaches.

**Additional file 3** Common Signature Genes and Enrichment Results Description: These are the PANTHER GO analysis results for the set of genes found to be in common among all data-derived signatures.

## Data Availability

The data sets involved in this study include the following previously published experiments hosted by GEO: GSE17721, GSE18921, GSE5099, GSE8286, GSE111475, GSE116999, GSE51587, GSE54017, GSE29797, GSE112899, GSE60235, GSE73213, GSE111789, GSE140443, GSE110446, GSE24791, GSE63038, GSE87290. GSE22248, GSE9916, GSE101710, GSE110223, GSE21045. The DC tolerogenicity signature data set was provided by Dr. Tatjana Nikolic upon request.

## Abbreviations

AUC: Area under the curve
DC: Dendritic cell
DE: Differential expression
DEG: Differentially expressed gene
EMBL: European molecular biology laboratory
GEO: Gene expression omnibus
GO: Gene ontology
GSA: Gene set analysis
GSEA: Gene set enrichment analysis
IL: Interleukin
KEGG: Kyoto encyclopedia of genes and genomes
LPS: Lipppolysaccride
mDC: Mature inflammatory dendritic cell
NCBI: National center for biotechnology information
NK: Natural killer
ORA: Over-representation analysis
PBMC: Peripheral blood mononuclear cell
ROC: Receiver operating characteristic
tol-DC: Tolerogenic dendritic cell
